# Eye Blink-Associated Saccades

**DOI:** 10.7759/cureus.18105

**Published:** 2021-09-19

**Authors:** Angela M Richmond, Blake D Sarrazin, Junaid H Siddiqui

**Affiliations:** 1 Neurology, University of Missouri, Columbia, USA; 2 Radiology, Gundersen Lutheran Medical Foundation, La Crosse, USA; 3 Neurology, Cleveland Clinic Foundation, Cleveland, USA

**Keywords:** saccadic eye movement, saccadic intrusion, movement disorders and tremors, eye blink, eye movements, middle cerebral artery infarction, drug-resistant epilepsy, temporal lobectomy

## Abstract

Saccades function to bring targets of interest into the field of view. They are one of the four types of basic eye movements in humans, all of which are generated and modulated by components of a complex eye movement network, involving cortical eye fields, thalami, basal ganglia, cerebellum, and brainstem structures. Similarly, blinks are presumed to be generated by a blink center involving complex cortical and subcortical pathways. An association between saccades and blinks is well established; when these circuits are disrupted, normal saccadic parameters change. We report a case of a 48-year-old female who presented with fatigue and weakness. She had a complicated medical history, including drug-resistant epilepsy with subsequent vagus nerve stimulator (VNS) placement, right anterior temporal lobectomy, and craniotomy for a residual right temporal lobectomy and amygdalohippocampectomy. The latter was complicated by ischemic right middle cerebral artery (MCA) territory stroke with residual left hemiplegia. Her examination was unremarkable with regards to the presenting complaints, but one unique finding was observed; she demonstrated abnormal conjugate eye movements to the left associated with each blink. These eye movements continued to be present even after the patient’s ability to fixate on an object was removed. It was unclear how long this finding had been present. A review of her MRI of the brain from 10 months prior showed encephalomalacia and surrounding gliosis in the right MCA territory, right temporal laminar necrosis, right basal ganglia and parietal lobe microhemorrhages, ex vacuo dilatation of the right lateral ventricle, and a rightward midline shift. Saccadic abnormalities have been reported in a variety of conditions. The eye blink-associated saccades seen here are rare. To our knowledge, only one other patient has been reported with similar blink-associated eye movements after brain injury following a right MCA territory stroke. The exact mechanism underlying these eye movements is unclear, but may involve aberrant or disrupted neuronal signaling in cortical and/or basal ganglia components of the eye movement network, or related to an as yet unknown blink-saccadic regulatory mechanism.

## Introduction

Saccades are rapid eye movements that function to bring targets of interest into a person’s field of view. They are one of four types of basic eye movements in humans, all of which are produced and modulated by components of a complex eye movement network, involving cortical eye fields, thalami, basal ganglia, cerebellar, and brainstem regions [1]. Blinks are also neural in origin and are thought to be generated by a blink center, which involves pathways between the cortex, subcortex (basal ganglia), brainstem, and visual and auditory systems [2]. An association between saccades and blinks is well established in normal brain function, as blinks influence saccadic parameters [3]. This association has been emphasized in patients with neurologic disorders in which there is a disruption in the blink and oculomotor circuits and is manifested as a change in saccadic features [4-5]. Here we describe a case of abnormal blink-associated saccadic-type conjugate eye movements in a middle-aged female with a history of a right temporal lobectomy and right MCA territory stroke. 

## Case presentation

**Figure 1:**
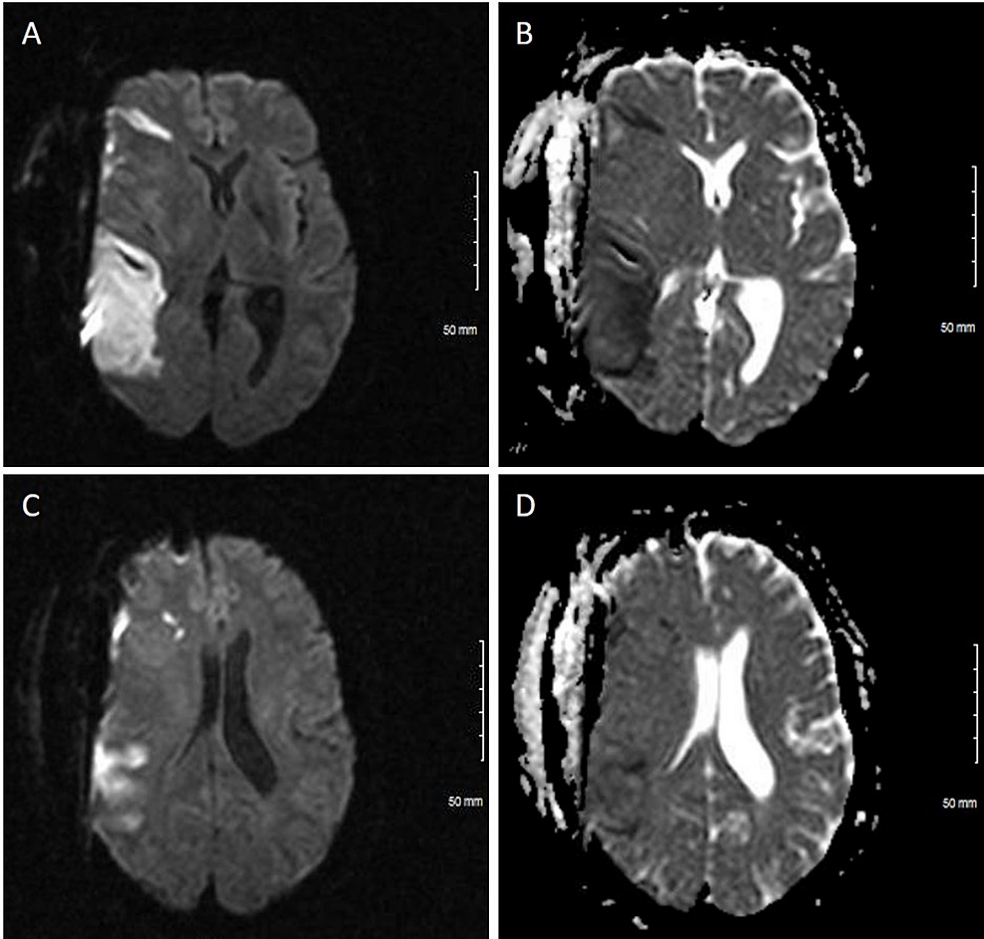
Brain MRI of the patient 36 hours after stroke symptoms Axial diffusion-weighted imaging (A, C) and apparent diffusion coefficient (B, D) MR sequences demonstrate evidence of an acute ischemic stroke in the right middle cerebral artery territory. Scattered foci of infarct are seen in the right frontal lobe and right posterior parietal lobe in two different planes (A-B, inferior; C-D, superior).

A 48-year-old female presented to the emergency department due to concern for fatigue, weakness, and delayed reactions. Her mother was her primary caregiver and described that the patient had been functioning at a level lower than her baseline status. She was unable to participate as usual in physical therapy the day prior due to “lifelessness” and appeared more tired than normal. The patient’s past medical and surgical history was complicated. She had a history of complex partial seizures sometimes with secondary generalization since age three. Etiology remained unknown, as there was no known history of brain injury or trauma, the patient met all of her developmental milestones on time, and was mainstreamed throughout her schooling years with infrequent seizures. During her late teenage years, she was seizure-free on phenobarbital and phenytoin and graduated from high school on time and without difficulty. However, her seizures returned a few years later, and they became increasingly resistant to multiple medications as she aged, requiring placement of a VNS at age 32 in addition to her medication regimen of four anti-epileptic drugs (AEDs). For a short time, the patient's seizure frequency improved, but by age 34, the frequency had increased so much despite maximal medical therapy and trialing more than 10 AEDs that the patient underwent right anterior temporal lobectomy. At ages 39 and 44, she underwent an uncomplicated replacement of her VNS battery. At age 46, her seizure frequency increased again, with multiple nocturnal seizures. Approximately one year later, at age 47, she underwent craniotomy for invasive EEG monitoring with subdural grid placement, followed by residual anterior-superior temporal lobectomy and frontal corticectomies. Her hospital course was complicated by a perioperative ischemic stroke in the right MCA territory, with scattered foci of infarct in the right frontal lobe and right posterior parietal lobe seen on MRI (Figure 1). This manifested as increased somnolence, left-sided hemiplegia, including left facial weakness, left-sided hypoesthesia, left-sided hemineglect, and left-sided hemianopsia. The etiology of her stroke was felt to be due to arterial thrombosis in the right MCA territory secondary to operative cortical vessel injury. Her clinical status improved substantially over the next two weeks but, on discharge, the patient required 24/7 care as she had a residual left-sided flaccid weakness that necessitated a wheelchair for ambulation. On the day of presentation, the patient's mother confirmed that the patient had been participating in routine physical therapy since her stroke 1.5 years prior and was receiving botulinum toxin therapy at regularly scheduled neurology appointments for left upper extremity spasticity. Her vision deficits had resolved, with no evidence of visual field deficits or hemineglect on subsequent exams. She had remained seizure-free since her last procedure. The inpatient neurology service was consulted to evaluate her for the decreased functional status.

The patient stated that although she was tired the day prior to the presentation, she was now back to her baseline. Her mother reported that the patient had seen her neurologist in the clinic two days before this visit; her escitalopram was discontinued and duloxetine started instead for migraine headaches and depression. The remainder of her medications were unchanged, including her nortriptyline for headache prevention and her antiepileptic drugs (lacosamide 250 mg bid, clobazam 15 mg bid, brivaracetam 100 mg bid, gabapentin 100 mg bid, lorazepam 1 mg tid as needed for two breakthrough seizures within a 24 hour period. bid: *bis in die* or twice a day, tid: *ter in die* or thrice a day). A complete review of systems was performed and was negative. On examination, she was alert and oriented with no evidence of change in her mental status from baseline. Her strength was 4+/5 in the right upper and right lower extremity. Her strength was 0/5 in her left upper and left lower extremity, with spasticity present in her left upper extremity, unchanged from her baseline.

However, one new finding was present; she was noted to have abnormal, fast conjugate eye movements to the left that were associated with each blink (Video 1). These eye movements continued to be present even after the patient’s ability to fixate on an object was removed with the use of Frenzel goggles (Video 2). The patient denied any subjective changes in her vision. Her pupillary responses were intact and she had normal visual acuity. She exhibited normal extraocular movements. It was unclear how long this finding had been present as the patient and her mother were unaware that these eye movements were happening and had not noticed them previously.

**Video 1:** The patient demonstrates abnormal, fast conjugate eye movements to the left that are associated with each blink.

**Video 2:** The same abnormal, fast conjugate eye blink-associated movements to the left exist despite removal of visual fixation with Frenzel goggles.

**Figure 2:**
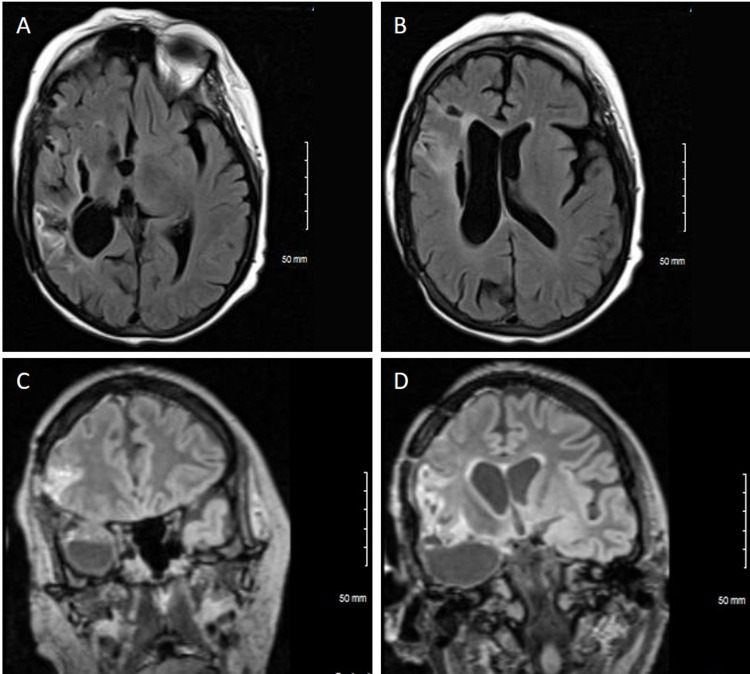
Brain MRI of the patient eight months after right middle cerebral artery territory stroke Axial (A, B) and coronal (C, D) T2/FLAIR MR sequences demonstrate encephalomalacia and surrounding gliosis in the right middle cerebral artery territory, including the right corona radiata. Also seen is right temporal laminar necrosis, ex vacuo dilatation of the right lateral ventricle, and interval development of a rightward 5 mm midline shift secondary to chronic right cerebral convexity volume loss. Images shown in two different planes each (A, inferior; B, superior; C, anterior; D, posterior).

The patient's laboratory workup was normal. Review of her most recent MRI of the brain completed 10 months prior (eight months post-stroke) showed encephalomalacia and surrounding gliosis in the right MCA territory, including the right corona radiata, with right temporal laminar necrosis, microhemorrhages in the right basal ganglia and right parietal lobe, ex vacuo dilatation of the right lateral ventricle with Wallerian degeneration of the right cerebral peduncle, and interval development of a rightward midline shift secondary to the chronic right cerebral convexity volume loss (Figure 2). The change in functional status the day prior was attributed to the patient's recent medication changes. Ultimately, she was deemed safe for discharge home with no changes to her medications. Since this consult, the patient has been examined numerous times at follow-up visits for management of her epilepsy and spasticity and continues to demonstrate the same abnormal blink-associated conjugate eye movements despite discontinuation of her duloxetine.

## Discussion

Saccades are rapid conjugate eye movements that shift the eyes to bring an object of interest into focus on the retinal fovea [6]. When they occur in response to the appearance of visual targets in the peripheral visual fields or auditory inputs, they are called reflexive. Alternatively, they are called intentional, or voluntary, when they are generated in response to command. Saccades can also occur spontaneously while at rest or during other activities. When saccades occur while patients are fixating on a target in the eyes' primary position, taking the fovea off the target, they are termed intrusions [6]. The abnormal conjugate eye movements observed in our patient occurred each time the patient blinked, regardless of which direction she was looking when she blinked. They also persisted despite the use of Frenzel goggles, which removes the wearer's ability to fixate on an object. The patient denied any sort of peripheral stimulus to her left or right. These findings support the idea that these abnormal eye movements are saccades, and are directly related to the patient's eye blinks. 

Eye movements are, in part, controlled by the frontal eye fields. The superior frontal eye field (sFEF) is located bilaterally at the intersection of the precentral sulcus and the superior frontal sulcus, although there has been significant variability in mediolateral positioning between individuals on positron emission tomography (PET) imaging [1,7-8]. It has numerous connections to other brain regions involved in oculomotor control [9-10]. Therefore, it is not surprising that the sFEF has been shown to be involved in all types of eye movements, including maintenance of fixation and preparation and initiation of voluntary saccades [11-14]. The inferior frontal eye field (iFEF) is located bilaterally in the dorsal branch of the inferior precentral sulcus [7]. Its exact function is less well understood, although it has been proposed that it may play a role in reflexive eye movements [14-15].

Eye movement abnormalities have been reported in a variety of conditions. One such example is the spasticity of conjugate gaze or lateral conjugate deviation of the eyes on the attempted forced closure of the lids. This phenomenon has been demonstrated in brains with unilateral cerebral injury, albeit rare [16]. The deviation is classically towards the side contralateral to the lesion. There are several hypotheses that attempt to explain this phenomenon, including suppression of the fixation reflex when the lids are closed or eyes covered, hypertonicity of the conjugate oculomotor mechanism contralateral to the lesion, and decreased ability to suppress attention in the contralateral visual hemifield when the eyes are closed [17-18]. The abnormal eye movements in our patient are directed to the left, contralateral to her brain injury. However, her eye movements occur spontaneously rather than on forced lid closure, persist despite removal of the patient’s ability to fixate on an object, and occur in the absence of an underlying gaze deviation, suggesting a mechanism independent from spasticity of conjugate gaze. Eye movement abnormalities have also been associated with medications. Duloxetine, a dual serotonin and norepinephrine reuptake inhibitor (SNRI), is known to impact eye movements, resulting in cycloplegia due to paralysis of the ciliary muscle, as well as symptoms of rapid eye movement sleep disorder (RBD). Although the patient was started on this medication only two days prior to presentation, she had no other eye movement abnormalities other than these eye blink-associated saccades, which have persisted for more than one year despite discontinuation of this medication. 

Like eye movements, blinks are thought to be regulated by a blink center that functions in parallel to the saccade and vergence systems [2]. Interestingly, activation of the iFEF has been shown to occur during blinks [1,7,19]. Studies have suggested that the blink center involves a complex pathway predominantly controlled by the extrapyramidal system and the superior colliculus, and is modulated by dopamine signaling [17]. The latter is supported by the observation that blink rate and saccade speed are decreased in Parkinson’s disease patients [20]. The blink center receives input from both cortical and brainstem structures such as the auditory and visual systems, and the pontine and medullary blink premotor areas, respectively, then projects to the facial nucleus and oculomotor nucleus, which control the orbicularis oculi and levator palpebrae muscles [2,18]. 

The abnormal blink-associated conjugate eye movements observed in this case are rare. To our knowledge, only one other patient has been reported with similarly described blink-associated conjugate eye movements after brain injury, which was following a right MCA territory stroke. Our case shares similarities with the case reported by Zivi, et al., including the presence of these blink-associated conjugate saccadic movements in the presence of otherwise intact oculomotor control, lack of gaze deviation, and evidence of a right MCA territory infarct without direct brainstem involvement on imaging [17]. We suspect that like the Zivi study [17], the abnormal eye movements seen in our patient developed sometime after her ischemic infarct, as these were never observed in the 14 years prior exam, after her first surgery, although we can not be sure. Moreover, on reviewing the images of our patient and the images of the patient described by Zivi et al., there is some overlap with regards to location of the injury in the right cortex, right corona radiata, and basal ganglia, the former of which may include the iFEF and/or circuits between this region and other cortical and subcortical areas necessary for motor planning and movement [1,17]. While the exact mechanism underlying these eye movements is unclear, it likely involves aberrant or disrupted neuronal signaling in specific cortical and/or basal ganglia components of the complex eye movement network and supports the possibility of an as yet unknown blink-saccadic circuit.

## Conclusions

Saccades are one of four types of eye movements, which are generated and modulated by components of an elaborate eye movement network, including cortical eye fields, thalami, basal ganglia, cerebellar, and brainstem structures. Abnormalities in vision, oculomotor control, and blinks have been demonstrated in a variety of neurological conditions. Astute assessment of eye movements during the neurological exam may shed light on the intricacies of this network, and provide a better understanding of the relationship between the brain regions, circuitry, and regulatory mechanisms involved in brain injury and disease. 

## References

[1] Coiner B, Pan H, Bennett ML (2019). Functional neuroanatomy of the human eye movement network: a review and atlas. Brain Struct Funct.

[2] Bour L, Ongerboer de Visser B, Aramideh M, Speelman J (2002). Origin of eye and eyelid movements during blinking. Mov Disord.

[3] Helmchen C, Rambold H (2007). The eyelid and its contribution to eye movements. Dev Ophthalmol.

[4] Hain TC, Zee DS, Mordes M (1986). Blink-induced saccadic oscillations. Ann Neurol.

[5] Barton JJ (1995). Blink- and saccade-induced seesaw nystagmus. Neurology.

[6] Kennard C, Crawford TJ, Henderson L (1994). A pathophysiological approach to saccadic eye movements in neurological and psychiatric disease. J Neurol Neurosurg Psychiatry.

[7] Amiez C, Petrides M (2009). Anatomical organization of the eye fields in the human and non-human primate frontal cortex. Prog Neurobiol.

[8] Pierrot-Deseilligny C, Milea D, Müri RM (2004). Eye movement control by the cerebral cortex. Curr Opin Neurol.

[9] Tomassini V, Jbabdi S, Klein JC (2007). Diffusion-weighted imaging tractography-based parcellation of the human lateral premotor cortex identifies dorsal and ventral subregions with anatomical and functional specializations. J Neurosci.

[10] Umarova RM, Saur D, Schnell S (2010). Structural connectivity for visuospatial attention: significance of ventral pathways. Cereb Cortex.

[11] O'Driscoll GA, Wolff AL, Benkelfat C, Florencio PS, Lal S, Evans AC (2000). Functional neuroanatomy of smooth pursuit and predictive saccades. Neuroreport.

[12] Matsuda T, Matsuura M, Ohkubo T (2004). Functional MRI mapping of brain activation during visually guided saccades and antisaccades: cortical and subcortical networks. Psychiatry Res.

[13] Heide W, Binkofski F, Seitz RJ, Posse S, Nitschke MF, Freund HJ, Kömpf D (2001). Activation of frontoparietal cortices during memorized triple-step sequences of saccadic eye movements: an fMRI study. Eur J Neurosci.

[14] Neggers SF, Diepen RM, Zandbelt BB, Vink M, Mandl RC, Gutteling TP (2012). A functional and structural investigation of the human fronto-basal volitional saccade network. PLoS One.

[15] McDowell JE, Dyckman KA, Austin BP, Clementz BA (2008). Neurophysiology and neuroanatomy of reflexive and volitional saccades: evidence from studies of humans. Brain Cogn.

[16] Smith JL, Gay AJ, Cogan DG (1959). The spasticity of conjugate gaze phenomenon. Arch Ophthalmol.

[17] Zivi I, Bertelli E, Bilotti G, Clemente IA, Saltuari L, Frazzitta G (2017). Blink-associated contralateral eccentric saccades as a rare sign of unilateral brain injury. Neurology.

[18] Rucker JC (2011). Normal and abnormal lid function. Handb Clin Neurol.

[19] Kato M, Miyauchi S (2003). Human precentral cortical activation patterns during saccade tasks: an fMRI comparison with activation during intentional eyeblink tasks. Neuroimage.

[20] Pretegiani E, Optican LM (2017). Eye movements in Parkinson's disease and inherited Parkinsonian syndromes. Front Neurol.

